# A computational method for drug repositioning using publicly available gene expression data

**DOI:** 10.1186/1471-2105-16-S17-S5

**Published:** 2015-12-07

**Authors:** KM Shabana, KA Abdul Nazeer, Meeta Pradhan, Mathew Palakal

**Affiliations:** 1 Department of Computer Science and Engineering, National Institute of Technology Calicut, Calicut, India; 2 IU School of Informatics and Computing, Indiana University Purdue University Indianapolis, Indianapolis, IN, USA

**Keywords:** drug repositioning, computational drug discovery, gene expression data

## Abstract

**Motivation:**

The identification of new therapeutic uses of existing drugs, or drug repositioning, offers the possibility of faster drug development, reduced risk, lesser cost and shorter paths to approval. The advent of high throughput microarray technology has enabled comprehensive monitoring of transcriptional response associated with various disease states and drug treatments. This data can be used to characterize disease and drug effects and thereby give a measure of the association between a given drug and a disease. Several computational methods have been proposed in the literature that make use of publicly available transcriptional data to reposition drugs against diseases.

**Method:**

In this work, we carry out a data mining process using publicly available gene expression data sets associated with a few diseases and drugs, to identify the existing drugs that can be used to treat genes causing lung cancer and breast cancer.

**Results:**

Three strong candidates for repurposing have been identified- Letrozole and GDC-0941 against lung cancer, and Ribavirin against breast cancer. Letrozole and GDC-0941 are drugs currently used in breast cancer treatment and Ribavirin is used in the treatment of Hepatitis C.

## Background

Despite the enormous investments in basic science and technology, the number of approved drugs reaching the market has been declining since the late 1990s. Bringing a new drug to market typically takes about 10 to 15 years and costs between $500 million and $2 billion [1].

If new uses can be identified for existing drugs, it can save both money and time, and improve treatments. In this context, the concept of drug repositioning is increasingly gaining importance. Drug repositioning is the process of identifying new indications for approved drugs. Apart from cheaper and faster drug development and reduced risks in drug discovery, drug repositioning offers several other merits. The new potential uses identified as a part of this process, which are not consistent with known disease mechanisms, might generate hypotheses that could lead to the discovery of new biological processes or disease pathways [1]. Drug repositioning can also lead to significant contributions in orphan drug development [2].

In the past, drug repositioning has often been accidental. There are many examples of repurposed drugs whose additional indications were discovered serendipitously. Another form of repurposing is the off-label use of medicines to treat a condition other than for which the drug was approved by FDA [1]. Post marketing surveillance information, including voluntary report by individual patients and physicians, can aid drug repositioning in a big way. Increased consumer activism, access to genetic information and social networking technologies are creating many opportunities for drug repositioning [1].

A number of computational approaches have been proposed to hypothesize which drugs from one disease indication can be used for another disease and they mainly fall into two categories, based on the data sources utilized [3]. The methods in the first category make use of certain static prior information, such as the target set of the drug and the structural and functional information of the target protein. This information is combined and utilized with different approaches for predicting new indications for drugs. Traditionally, the idea of drug repositioning has been based on understanding how the drug interacts with various pathways in specific cells in the body [4]. These methods try to identify diseases with similar structures or molecular alterations that could benefit from the same drug.

The methods in the second category make use of microarray data to represent cellular state and reposition drugs against various diseases [3]. Methods under this category follow the common assumption that gene expression of many diseases and drugs can characterize to some extent the effects of diseases and drugs and therefore they can be related based on the similarity/dissimilarity of their expression profiles [5]. Ideally the interference of the drug should restore the cellular state to normal state and the changes of the transcriptional level induced by the drug should reverse the changes in the transcriptional level under disease state. Thus the basic idea is that a drug will have the potential to cure a disease if the differential expression profile under drug administration and disease states is anti-correlated significantly [3].

## Related work

Butte et al. [6] combined data from publicly available microarray data sets representing 100 diseases and gene expression data from human cell lines treated with 164 drugs or small molecules, obtained from Connectivity Map [7], to predict therapeutic drug-disease interactions. They generated genome-wide mRNA signatures for drug treated cell lines and also calculated signatures from various disease states. Each of the disease signatures was statistically compared to each of the reference drug expressions from the Connectivity Map and a similarity score was calculated for every pairing of drug and disease reflecting the similarity of the drug and disease signatures [6]. Using the hypothesis that drug-cell signatures that anticorrelated with disease signatures could be of therapeutic value [4], they recovered many known drug and disease relationships and predicted many new indications for approved drugs.

Iorio et al. [8] used similarity in gene expression profiles following drug treatment, across multiple cell lines and dosages, to predict similarities in drug effect and mode of action. For each drug, a consensus transcriptional response was developed summarising the transcriptional effect of drug across multiple treatments. A drug network was constructed next in which two drugs are connected if their consensus responses are similar. By analyzing the interconnected modules, similarities and differences in pharmacological effects and modes of action were predicted.

A large-scale disease-disease, drug-drug and disease-drug network was generated by Guanghui and Agarwal [5] by directly matching their transcriptomic profiles obtained from human Gene Expression Omnibus(GEO) [9] data sets. Human GEO data sets were used to generate disease and drug genomic profiles. The links between different diseases and drugs were established using two different methods. The first method was based on the concept of *correlation*, which measures profile-profile similarity, whereas the second method, based on the concept of *enrichment*, measures the signature-profile similarity. The disease-disease network provided a new way to redefine human diseases and gain a broader understanding of the disease mechanism. The connected diseases that are located in different branches of MeSH tree provided potentially novel disease relationships. The genomic profile-based disease relationship helped in drug repositioning. If two diseases are linked in a sub-network, then this indicates that the diseases may be similar and hence the drug used for one disease may be repurposed for the other.The disease-drug sub-networks were used to generate hypotheses on the potential drug side effects and perform drug repositioning. Among the disease-drug links, connections with negative scores suggested new indications for existing drugs, while the positive scoring connections could aid in drug side effect elimination.

A new method for identifying potential drugs for repositioning was proposed by Zikai et al. [3] by introducing a new measure considering both efficiency and side effect. The cellular network is a complex networked system and hence the effect on some cellular elements induced by the drug will propagate through the network. Therefore the drug can induce both the desired effect and some unintended effect simultaneously. The number of abnormally regulated genes after drug treatment that were regulated oppositely under disease state was used to measure the efficiency of the drug. Certain genes which are newly regulated or further regulated in the same direction after drug treatment were considered the source of side effect. The changes in transcription level of essential genes, which are indispensable to support cellular life, might cause significant unfavourable phenotype variation, such as side effect. Therefore, the number of essential genes that were newly regulated or further regulated in the same direction after drug treatment was used to measure the extent of side effect. Based on these two measures, a new scheme was developed to score and rank drug-disease associations and reposition drugs.

## Methods

We propose a novel methodology to perform drug repositioning without using gene signatures. In this method, *normal plus disease *data set and *pre and post drug treatment *data set associated with a disease are used. The *normal plus disease *data set contains gene expression data of samples in normal and disease states. The *pre and post drug treatment *data set contains gene expression data of samples before and after treatment with a particular drug. The significant genes associated with the disease are determined by processing the *normal plus disease *data sets. Also the genes affected by the drug are identified by processing the *pre and post drug treatment *data sets. Next by comparing the two sets, the disease genes targeted by the drug are identified. Hyperedges are then constructed for each drug, the analysis of which leads to the identification of the drugs that target genes causing various diseases.

The proposed method has been outlined below:

**Input**: The *normal plus disease *data set, and *pre and post drug treatment *data set associated with two cancerous and two non-cancerous diseases

**Output**: A list of drugs that can be used to treat genes causing different diseases

Step 1: Process the *normal plus disease *data set to identify the significant genes, Set A

Step 2: Similarly process the *pre and post drug treatment *data set to create a Set B containing significant up and down regulated genes. The disease genes that exhibited up/down regulation under the administration of at least one drug are considered for further analysis.

Step 3: Compare the genes of Set A and Set B for the same disease. If the genes are common and they are opposite i.e. in Set A, an identified significant gene is up-regulated and after treatment in Set B, this gene is down-regulated, or vice versa, then this implies that the gene is a probable target of the drug

Step 4: Construct disease networks for each disease using the identified significant genes and their interactions. Compute their node weights and edge weights

Step 5: Construct a single network comprising of all the disease genes along with nodes for each disease

Step 6: Construct hyperedges in this network for each drug. Analysis of genes in this hyperedge can yield novel disease-gene-drug relations

Network I consists of gene-gene interactions (Set A and Set B). A separate network is constructed for each disease. The node weight, edge weight and node strength are computed as follows:

(1) Nodeweight=Normalized(degree)+Normalized(betweenness)+Normalized(clustering coefficient)

(2) $$\text{Edge}\;\;\text{weight}=Average\left(\text{Gene}\;\;\text{Ontology}\;\;\text{Semantic}\;\;\text{Similarity,Pathway}\;\;\text{Score}\right)$$

(3) $$\text{Pathway score}=\frac{\text{Number}\;\;\text{of}\;\;\text{common}\;\;\text{pathways}\;\;\text{across}\;\;\text{the}\;\;\text{two}\;\;\text{nodes}}{\text{Total}\;\;\text{number}\;\;\text{of}\;\;\text{pathways}\;\;\text{of}\;\;\text{the}\;\;\text{two}\;\;\text{nodes}}$$

(4) $$\text{Node}\;\;\text{strength}=\text{Node}\;\;\text{weight}+\Sigma \left(\text{Edge}\;\;\text{weight}\;\;\text{of}\;\;\text{all}\;\;\text{incident}\;\;\text{edges}\right)$$

Network II consists of gene-gene interactions (Set A and Set B) together with disease-gene interactions. It is a single network comprising of all the disease genes along with separate nodes for each disease. Each disease node shares an edge with each of its significant disease genes. The node weight of the gene nodes and the edge strengths are the same as in Network I. For the disease nodes, the node weight is calculated in the same way as it was done for genes in Network I. The disease- gene edge weight is calculated by computing the z-score of the node weights of all significant disease genes. Next the genes are ranked based on the z-score and the disease-gene edge weight is calculated as:

(5) $$\text{Edge Weight(D,G) }=\;1-\frac{rank\left(G\right)}{N}$$

where N is the total number of significant genes associated with disease D

The drug nodes are then added to Network II and each drug node is connected to the genes which show opposite regulation under its administration. Hyperlinks are observed where both disease-gene and drug-gene edges are incident on a single gene node, representing a disease-gene-drug relationship. All the gene nodes that are connected to each drug node along with the disease nodes form a hyperedge. These hyperedges are used to perform drug repositioning.

The hyperedge associated with each drug consists of all the disease nodes and the gene nodes associated with the drug. The adjacent genes of the disease nodes in the hyperedge are identified to find out the genes associated with each disease that are affected by the drug. The set of genes thus identified for each disease is further refined by selecting only the ones that become oppositely regulated under the administration of the drug. Thus for each disease, we get a set of gene nodes that are targeted by the drug. The node strength of these genes in the disease network determine the prospects of repurposing the drug against the disease.

The disease-gene-drug connections are ranked based on a scoring function and an overall score is also assigned for each disease-drug network. The scoring function of disease-gene-drug takes into account the relative contribution of the gene in the disease (A) and also the relative effect of the drug on the gene (B). Here it is assumed that the drug has equal effect on each of its target genes. The scoring function is the product of the parameters A and B, as given below:

(6) $$\text{Score}\left(\text{Ds,G,Dr}\right)=A*B$$

(7) $$A=\frac{P}{Q}$$

(8) $$B=\frac{1}{S}$$

P: Node weight of gene G in network of Ds

Q: Sum of node weights of all the genes associated with disease Ds

S: Number of genes associated with Ds which are affected by drug Dr

In order to score the disease-drug connection, the sum of the node weights of the genes, affected by the drug, in the disease network and the sum of the node weights of all the genes in the disease network are taken into account. It gives a measure of the efficiency of the drug on the disease in terms of the relevance of the target genes in the disease network. The scoring function for the disease-drug connection is defined as follows:

(9) $$Score\left(Ds,Dr\right)=\frac{Y}{Z}$$

Y: Sum of node weights of all genes associated with Ds and affected by Dr

Z: Sum of node weights of all genes associated with Ds

The disease-drug score, Score(Ds,Dr), is the proportion of node weights in the disease network of Ds targeted by Dr. A high disease drug scores indicate that the drug targets a good proportion of genes in the disease network. But even if the scores are low, Dr can be a potential candidate for repurposing if it is found to target some of the important biomarkers of the disease Ds.

## Implementation

The gene expression data associated with *normal plus disease *and *pre and post drug treatment *data sets for two cancerous diseases- Lung Cancer and Breast Cancer and two non-cancerous diseases- Parkinson's Disease and Hepatitis C were collected from the website of National Center for Biotechnology Information (NCBI) GEO database [9]. The raw data was downloaded and then normalized using the Robust Multichip Average (RMA) [10] normalization procedure in the R [11] package called affy [12]. The data sets were annotated with the latest corresponding GEO Platform (GPL) annotation file downloaded from the website of AILUN [13]. The probe ids were mapped to the corresponding Gene Symbol, if it exists. All the gene expression entries without a Gene Symbol were removed. In cases where multiple microarray probe sets mapped to the same Gene Symbol, their mean expression value was assigned to the Gene Symbol.

The differentially expressed genes were identified using the empirical bayes method with linear modelling approach [14] implemented in the R package called limma [15]. For the cancerous diseases, the FDR threshold of 0.01 for *q value *was used in *t−test *to identify the significant genes for both *normal plus disease *and *pre and post drug treatment *data sets. The FDR threshold of 0.05 for *q value *was used in the case of non-cancerous diseases. The number of significant genes identified for the cancerous and non-cancerous data sets have been given in Table 1. The log2 fold change of genes was used [16] to find whether the gene is up-regulated or down-regulated. If the log2 fold change value is positive, then the gene is up-regulated. Otherwise, it is down-regulated. For a given disease, if there were multiple data sets from the same platform, the common genes and unique genes were identified separately and the up/down regulation of these genes were noted.

**Table 1:** No: of Significant Genes Identified in Cancerous and Non-Cancerous Data sets.

Disease	GSE	No: of Genes
Lung Cancer	**Disease** GSE18842 GSE19188 GSE19804	8287 5792 4648
	**Drug** GSE6400 (Acitnomycin) GSE6400 (1.25uM Sapphyrin PCI-2050) GSE6400 (2.5uM Sapphyrin PCI-2050)	3990 4655 1755

Breast Cancer	**Disease** GSE10810 GSE26910	5887 102
	**Drug** GSE10281 GSE11352 (12 hr) GSE11352 (24 hr) GSE11352 (48 hr) GSE20719 GSE28305(16 hr) GSE28305(48 hr)	168 532 475 766 2441 23 176

Parkinson's	**Disease** GSE7621	40
	**Drug** GSE14429(1 hr) GSE14429(6 hr)	302 7668

Hepatitis C	**Disease** GSE38597	881
	**Drug** GSE23031	5421

The significant genes of *pre and post drug treatment *data sets were compared against the significant genes of the corresponding disease and the genes whose regulation has been made opposite under the administration of drug were identified. Multiple dosages of the same drug were treated separately, whereas in the case of application of the same drug across multiple time points, only the common significant genes across all the time points were taken into consideration.

The gene-gene interactions of the significant genes were obtained from BioGRID Version 3.2.97 [17]. The interactions obtained from both high and low throughput experiments were considered. The interactions were further filtered by considering only those in which both the interacting genes belong to the set of significant genes under consideration. The results are summarized in Table 2. After this step, it was observed that the number of interactions for Parkinson's Disease were very less. Hence Parkinson's Disease was dropped from the analysis.

**Table 2:** BioGRID Interactions Summary.

Disease	Significant No: of Disease Genes	No: of Interactions Involving Only Significant Genes
Lung Cancer	3980	11394
Breast Cancer	1468	1802
Hepatitis C	281	55
Parkinson's Disease	21	4

Disease network for each disease was constructed with the identified significant genes forming the nodes of the graph and the edges representing the interactions between genes obtained from BioGRID. Network II, the single network comprising of all the disease genes along with nodes associated each disease, was also created. It is a single network comprising of all the disease genes along with separate nodes for each disease. Each disease node shares an edge with each of its significant disease genes. The node weights and edge weights of all the graphs were computed using equations 1, 2 and 5.

The construction and analysis of graphs was performed using the igraph [18] package of R. The Gene Ontology (GO) score for a pair of genes was computed based on Wang's method[19] using the R package GOSemSim [20]. The pathway information about genes was obtained from KEGG [21].

**Figure 1:**
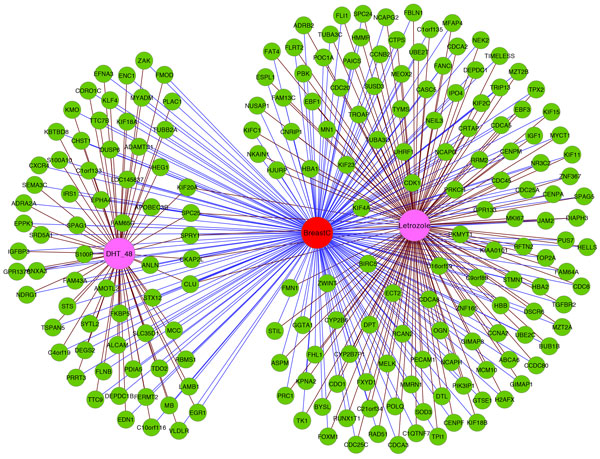
**HyperLinks in Network II of Breast Cancer on adding Drug Nodes associated with Letrozole and 5aDHT**. The red color node is the disease node representing Breast Cancer and the purple nodes represent the drug nodes, namely 5aDHT and Letrozole. The green colored nodes are the gene nodes representing the genes associated with Breast Cancer. All gene nodes are connected to the disease node. The gene nodes are also connected to the drug nodes that make them oppositely regulated. The hyperlinks are observed where both disease-gene (blue) and drug-gene (magenta) edges are incident on a single gene node (green). All the gene nodes that are connected to both the disease node and the drug node Letrozole form one hyperedge. Similarly another hyperedge associated with the drug node 5aDHT can also be observed.

The hyperlinks formed in Network II on adding the drug nodes are shown in Figure 1. Here the central node in red color is the disease node corresponding to Breast Cancer. The two other central nodes in purple color are the drug nodes corresponding to 5aDHT and Letrozole. The other nodes colored green are the gene nodes representing the genes associated with Breast Cancer. All gene nodes are connected to the disease node. The gene nodes are also connected to the drug nodes that make them oppositely regulated. The hyperlinks are observed where both disease-gene (blue) and drug-gene (magenta) edges are incident on a single gene node (green). All the gene nodes that are connected to both the disease node and the drug node Letrozole form one hyperedge. Similarly another hyperedge associated with the drug node 5aDHT can also be observed.

**Figure 2:**
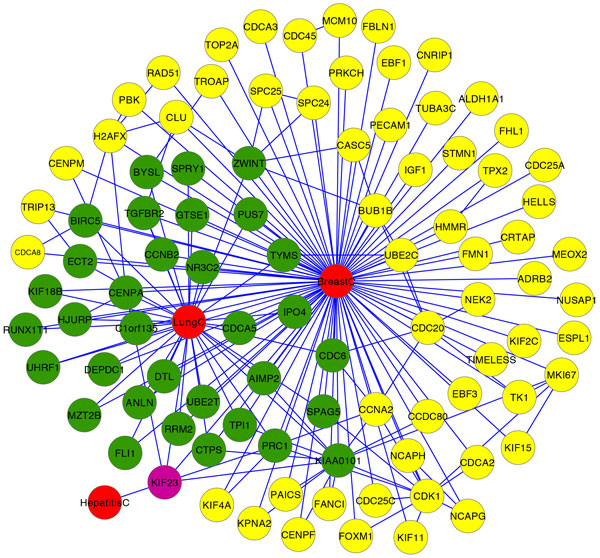
**Disease Nodes, Genes and their Connections in the Hyperedge of the Breast Cancer drug Letrozole.** The nodes colored in red are the disease nodes - Breast Cancer, Lung Cancer and Hepatitis C. The yellow nodes are the genes associated only with breast cancer. The nodes colored green represent the genes associated with both breast cancer and lung cancer and the purple color node denotes the gene associated with the two cancerous diseases as well as with Hepatitis C.

The nodes in the hyperedge associated with the Breast Cancer drug Letrozole is given in Figure 2. The nodes colored in red are the disease nodes. The yellow nodes are the genes associated only with breast cancer. The nodes colored green represent the genes associated with both breast cancer and lung cancer and the purple color node denotes the gene associated with the two cancerous diseases as well as with Hepatitis C.

The Disease-Drug scores for various drugs and diseases, computed using equation 9, have been summarized in Table 3.

**Table 3:** Summary of Disease-Drug Scores.

Drug	Breast Cancer	Hepatitis C	Lung Cancer
Letrozole	0.1593	0.0178	0.0138
GDC-0941	0.5652	0	0.0825
5a-DHT	0.0086	0	0.0017
Ribavirin	0.0160	0.5427	0.0118
Acitnomycin D	0.2231	0.0178	0.2851
Sapphyrin PCI-2050 (1.25uM)	0.0984	0.1624	0.3190
Sapphyrin PCI-2050 (2.50uM)	0.0685	0	0.1258

## Results and discussion

By ranking the genes based on their node strength in each of the disease networks, the significant genes targeted by the drug set and are highly related with each disease have been identified.

Out of the top 10 significant genes associated with breast cancer, 7 were validated using GeneCards, which is a compilation of annotative information about human genes, mined and integrated from over 80 digital sources [22]. In the case of lung cancer, 6 out of the top 10 were verified [22]. 3 genes out of the top 10 were found to be associated with Hepatitis C [22]. The top 10 genes identified for each disease have been listed in Table 4.

**Table 4:** The Top 10 Genes Identified for Breast Cancer, Lung Cancer and Hepatitis C.

Breast Cancer Genes	Lung Cancer Genes	Hepatitis C Genes
ESR1	UBC	TP53
HDAC5	ELAVL1	RAD21
BRCA1	CUL3	EWSR1
SMAD3	SIRT7	BAD
H2AFX	CAND1	CARM1
ARRB1	MYC	STK11
XPO1	EP300	WDR5
CCNA2	BRCA1	YY1
EIF4A3	PAXIP1	BTBD2
NOP56	SMAD3	EEF1D

In our analysis, it was found that the breast cancer drug Letrozole targeted some of the lung cancer genes. Out of the 34 target genes found to be associated with Lung Cancer, 17 were validated from literature [22]. In the light of the recent studies indicating that blocking estrogen is crucial in developing effective treatments for lung cancer [23], we analyzed the targets of Letrozole and found out several genes including RRM2, TOP2A and RAD51 that were estrogen responsive [24]. It has also been reported that Letrozole decreased cell proliferation in ER expressing cell lines in Non Small Cell Lung Carcinoma (NSCLC) [25]. In the light of these evidences, we propose Letrozole as a strong candidate for repurposing against lung cancer.

It can be seen from Table 3 that Letrozole has a higher score for Hepatitis C. This is because it targeted genes with higher node weights in the disease network of Hepatitis C as compared to Lung Cancer. Even though the sum of the node weights of the lung cancer genes targeted was less, (hence the lower score compared to Hepatitis C), it was verified from literature that some of the lung cancer genes targeted by Letrozole were significant in developing a treatment to the disease. Hence we proposed Letrozole as a more plausible candidate for repurposing against lung cancer as compared to Hepatitis C.

In the case of the drug GDC-0941, which has been found effective in treatment of breast cancer, we observed that the drug targeted 204 lung cancer genes and 52 of them were verified as being associated with lung cancer from literature [22]. Also four target genes, CCNE2, E2F3, TRAF2 and TRAF4 were identified as being part of the lung cancer disease pathway [21]. A study conducted in mice show that GDC- 0941 has excellent anti-tumor activity against various cancers [26]. Another study has shown that treatment with GDC-0941 led to pronounced tumor shrinkage and inhibition of tumor growth in two NSCLC models in mice [27]. Hence we suggest that GDC-0941 can be repurposed against lung cancer.

Ribavirin is a drug prescribed for Hepatitis C. In our analysis, it was observed that the drug targeted the gene KIF18A, which is a potential target for breast cancer [28]. Another potential target for triple negative breast cancer, LRP8 [29], was also found to be targeted by Ribavirin. Targeting MYC-regulated pathways has been proposed as a promising therapeutic strategy for breast cancer [30]. And in one of the recent studies, it has been reported that in breast cancer cell lines, MYC expression is dependent on the RAD21 subunit of cohesin [31]. It was observed in our analysis that Ribavirin targeted RAD21 by making it down-regulated. In the context of these evidences, we propose Ribavirin as a strong candidate for repurposing against breast cancer.

Acitnomycin D and Sapphyrin PCI-2050 are drugs used in the chemotherapy for lung cancer. It was observed that Acitomycnin D, 1.25uM Sapphyrin PCI-2050 and 2.50uM Sapphyrin PCI-2050 targeted 123, 114 and 47 breast cancer genes respectively, out of which 37, 34 and 13 genes respectively were validated from literature as being associated with breast cancer [22]. These 3 drugs targeted the gene E2F3, which has been identified as a potential therapeutic target in breast cancer [32]. Hence we suggest that Acitnomycin D and Sapphyrin PCI-2050 may be considered for repurposing against breast cancer.

5a-Dihydrotestosterone (DHT) is a steroid used in the treatment of breast cancer. This drug targeted 10 lung cancer genes and 3 of them were verified from literature [22]. So we propose 5a-DHT as a weak candidate for repurposing against lung cancer.

## Conclusions

We have proposed a new computational method to identify candidates for drug repositioning for breast cancer and lung cancer using gene expression data. This method can be extended to other diseases and drugs to identify novel therapeutic relationships. The effectiveness of the proposed method can be improved by including all the significant disease genes and their interactions rather than considering only the genes that are affected by a drug.

This method is based on the hypothesis that diseases and drugs can be related based on the similarity/dissimilarity of their gene expression profiles. Hence the validation of the proposed candidates for repurposing would require additional experiments in lab by the domain experts. It would be interesting to look at other ways to validate the plausible candidates for repurposing. The proposed method using hyperedges can be made scalable using graph databases such as HyperGraphDB, which is designed for complex, large scale knowledge representation applications such as the ones found in artificial intelligence, bio-informatics and natural language processing [33].

## Competing interests

The authors declare that they have no competing interests.

## Authors' contributions

MJP and MP introduced the problem and proposed the algorithm. KMS worked on data collection, implementation of the method and the analysis of the results. KAAN supervised the research and edited the manuscript. All authors read and approved the final manuscript.

## Declarations

Publication of this article was supported by the National Institute of Technology Calicut, Kerala State, India.

This article has been published as part of *BMC Bioinformatics *Volume 16 Supplement 17, 2015: Selected articles from the Fourth IEEE International Conference on Computational Advances in Bio and medical Sciences (ICCABS 2014): Bioinformatics. The full contents of the supplement are available online at http://www.biomedcentral.com/bmcbioinformatics/supplements/16/S17.
